# Ectopic expression of a novel *OsExtensin‐like* gene consistently enhances plant lodging resistance by regulating cell elongation and cell wall thickening in rice

**DOI:** 10.1111/pbi.12766

**Published:** 2017-07-15

**Authors:** Chunfen Fan, Ying Li, Zhen Hu, Huizhen Hu, Guangya Wang, Ao Li, Youmei Wang, Yuanyuan Tu, Tao Xia, Liangcai Peng, Shengqiu Feng

**Affiliations:** ^1^ Biomass and Bioenergy Research Centre Huazhong Agricultural University Wuhan China; ^2^ National Key Laboratory of Crop Genetic Improvement Huazhong Agricultural University Wuhan China; ^3^ College of Plant Science and Technology Huazhong Agricultural University Wuhan China; ^4^ College of Life Science and Technology Huazhong Agricultural University Wuhan China

**Keywords:** Lodging resistance, extensins, transgenic rice, cell wall, cell elongation, mechanical strength

## Abstract

Plant lodging resistance is an important integrative agronomic trait of grain yield and quality in crops. Although extensin proteins are tightly associated with plant cell growth and cell wall construction, little has yet been reported about their impacts on plant lodging resistance. In this study, we isolated a novel extensin‐like (OsEXTL) gene in rice, and selected transgenic rice plants that expressed *OsEXTL* under driven with two distinct promoters. Despite different *OsEXTL* expression levels, two‐promoter‐driven *OsEXTL*‐transgenic plants, compared to a rice cultivar and an empty vector, exhibited significantly reduced cell elongation in stem internodes, leading to relatively shorter plant heights by 7%–10%. Meanwhile, the *OsEXTL*‐transgenic plants showed remarkably thickened secondary cell walls with higher cellulose levels in the mature plants, resulting in significantly increased detectable mechanical strength (extension and pushing forces) in the mature transgenic plants. Due to reduced plant height and increased plant mechanical strength, the *OsEXTL*‐transgenic plants were detected with largely enhanced lodging resistances in 3 years field experiments, compared to those of the rice cultivar ZH11. In addition, despite relatively short plant heights, the *OsEXTL*‐transgenic plants maintain normal grain yields and biomass production, owing to their increased cellulose levels and thickened cell walls. Hence, this study demonstrates a largely improved lodging resistance in the *OsEXTL*‐transgenic rice plants, and provides insights into novel extensin functions in plant cell growth and development, cell wall network construction and wall structural remodelling.

## Introduction

Lodging is a major limiting factor of grain production by reducing photosynthetic ability and affecting grain filling in food crops (Weber and Fehr, 1966). Because high moisture in lodged plant communities is favourable for fungal growth and disease development, lodging not only affects grain quality and appearance, but also leads to a pre‐harvest germination (Kono, 1995). In addition, lodging largely reduces crop mechanical harvesting efficiency (Berry *et al*., 2004).

Lodging usually occurs while plant stems bend or break at the basal internode, and it is thus caused by a loss of balance within the plant (Pinthus, 1974). Plant lodging resistance is mainly determined by the weight of its upper portion (upper leaves, stems and seeds) and the pushing resistance of the lower portion (Mulder, 1954). Therefore, plant height is a main target for lodging resistance improvement. For example, the semi‐dwarf lines have been widely used as rice “green revolution” (Keller *et al*., 1999; Khush, 1999). Although several genes related to plant height have been identified, such as *RGA1* (Ashikari *et al*., 1999; Fujisawa *et al*., 1999), *OSH15* (Sato *et al*., 1999) and *sd‐1* (Monna *et al*., 2002; Sasaki *et al*., 2002; Spielmeyer *et al*., 2002), they have not yet been used in practical breeding, probably due to negative effects on grain yield and other important agronomic traits. For instance, seeds of the *RGA1*‐transgenic rice plants are much smaller than those of control plants (Ashikari *et al*., 1999), and the retrotransposon‐induced mutation of OSH15 reduces panicle length (Sato *et al*., 1999).

Many studies have been conducted on the correlation between stem characteristics and lodging resistance. Plant lodging resistance is positively correlated with either stiffness of the basal stems or cell wall thickness of stem tissues. For example, lodging‐resistant cultivars exhibit thicker culm walls than those susceptible to lodging (Islam *et al*., 2007; Kelbert *et al*., 2004; Tripathi *et al*., 2003; Zuber *et al*., 1999). In addition, contents of various biochemical components can also determine stem rigidity including cellulose, starch and sugars (Ishimaru *et al*., 2008; Kashiwagi and Ishimaru, 2004; Kashiwagi *et al*., 2006; Li *et al*., 2015; Somerville *et al*., 2004). In particular, brittle culm mutants identified in rice have largely reduced mechanical strength with decreased cellulose contents and altered cell wall structures (Li *et al*., 2011; Tanaka *et al*., 2003; Xiong *et al*., 2010; Zhang and Zhou, 2011; Zhang *et al*., 2010). Hence, it has been suggested that an improvement in lodging resistance could be achieved by reducing plant height and increasing stem stiffness.

Various parameters are used for evaluating lodging resistance, and three criteria are highly associated with visual score of lodging. As the most direct criteria indicate the degree of plant lodging resistance, the lodging index arises from the bending or breaking of the lower culm internodes and is highly related to plant height, fresh weight, stem diameter and others (Crook and Ennos, 1994; Islam *et al*., 2007). In addition, extension force is the second criterion, measuring the elasticity of the plant organs (Zhang *et al*., 2016). Finally, pushing force is the third criterion, measured while the plant is pushed to an angle of 45° from the vertical (Berry *et al*., 2004; Hai *et al*., 2005).

During plant stem growth, plant cell walls maintain their thickness through the addition of newly synthesized polysaccharides and proteins (Cosgrove, 2000). In growing plant tissues, however, the cell walls must possess sufficient tensile strength to resist the high turgor pressure that drives growth, while simultaneously remaining flexible enough to selectively yield and expand (Cosgrove, 2005; McCann and Roberts, 1994). In principle, plants produce two major types of cell walls: primary and secondary cell walls. The primary cell wall is an elastic structure that responds to the requirements of cell growth (Darley *et al*., 2001), whereas the secondary cell wall is a rigid, thickened structure that determines the mechanical strength of the plant body (Taylor *et al*., 2000). Hence, both types of cell walls have distinct structures composed mostly of polysaccharides, lignin and highly glycosylated proteins (Somerville *et al*., 2004).

Extensins are one of the major classes of hydroxyproline‐rich glycoproteins (HRGPs) present in plant cell walls (Chen and Varner, 1985; Lamport, 1963; Showalter, 1993). Extensins are characterized by the repeated occurrence of serine followed by proline residues (Kieliszewski and Lamport, 1994; Liu *et al*., 2016; Memelink *et al*., 1993). Previous studies have demonstrated that extensins are required for normal vegetative growth, male fertility and disease tolerance. Mutation of extensins genes leads to fewer leaves and reduced plant size and fertility in *Arabidopsis* (Cannon *et al*., 2008; Choudhary *et al*., 2015; Saha *et al*., 2013). Short and irregular root hair lengths are also observed in the insert mutations of *AtEXT6*,* 7*,* 10*,* 11*,* 12* and *13* (Velasquez *et al*., 2011). Despite reports showing that overexpression of extensin‐like genes affects stem height (Roberts and Shirsat, 2006), cell wall properties (Tan *et al*., 2014) and disease tolerance (Balaji and Smart, 2012; Wei and Shirsat, 2006), little has been reported about the role of extensins in lodging resistance in cereal crops. In this study, we first isolated a novel rice extensin‐like gene, and overexpressed it using two distinct promoters. We then determined largely enhanced lodging resistance in the transgenic rice plants, and proposed a model about its dynamical regulation on cell elongation and cell wall thickening for plant mechanical strength and lodging character.

## Results

### *OsEXTL* isolation and expression observation

**Figure 1:**
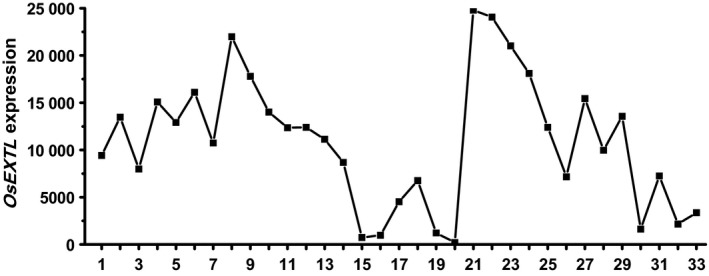
*OsEXTL* expression profiling in rice life cycle. The X‐axis indicates the tissues at the developmental stages: 1, Calli (15 days after subculture); 2, Calli (5 days after regeneration); 3, Calli (Screening stage); 4, Calli (15 days after induction *T*_2_); 5, Calli (15 days after induction *T*_3_); 6, Seed imbibition; 7, Seed germination; 8, Plumule (48 h after emergence, Dark); 9, Plumule (48 h after emergence, Light); 10, Radicle (48 h after emergence, Dark); 11, Radicle (48 h after emergence, Light); 12, Seedling; 13, Young shoot; 14, Young root; 15, Mature leaf; 16, Old leaf; 17, Mature sheath; 18, Old sheath; 19, Young flag leaf; 20, Old flag leaf; 21, Young panicle stages 3 (secondary branch primordium differentiation stage); 22, Young panicle stages 4 (pistil/stamen primordium differentiation stage); 23, Young panicle stages 5 (pollen‐mother cell formation stage); 24, Young panicle; 25, Old panicle; 26, Young stem; 27, Old stem; 28, Hull; 29, Spikelet; 30, Stamen; 31, Endosperm (7 days after pollination); 32, Endosperm (14 days after pollination); 33, Endosperm (21 days after pollination). The Y‐axis represents the *OsEXTL* relative expression levels obtained from microarray analysis.

Based on the rice genomic sequence database, a novel OsExtensin‐like (OsEXTL) gene was isolated in Zhonghua 11 (ZH11), a rice cultivar (*Oryza sativa* ssp. *japonica*). According to phylogenetic analysis (Figure S1), the OsEXTL is much farther away from total 27 well‐known OsExtensin proteins, and thus termed as OsExtensin‐like gene in this study. Using public expression profile data (Table S1) obtained from CREP database (http://crep.ncpgr.cn) (Wang *et al*., 2010), we observed *OsEXTL* gene expression pattern in almost all 33 tissues covering the entire life cycle of rice. In particular, relatively high expression of *OsEXTL* was found in tissues of calli, seed imbibition/germination, plumule and radicle, seedling, young shoot, young root, panicle, stem, hull, spikelet and stamen (Figure 1). By comparison, the *OsEXTL* showed exceptionally lower expression in the leaf and sheath tissues of mature rice. Hence, it indicated that the *OsEXTL* gene is mainly expressed in young tissues tightly associated with cell elongation and primary cell wall biosynthesis (Cosgrove, 1997).

### Selection of transgenic rice plants that expressed *OsEXTL* under driven with two promoters

**Figure 2:**
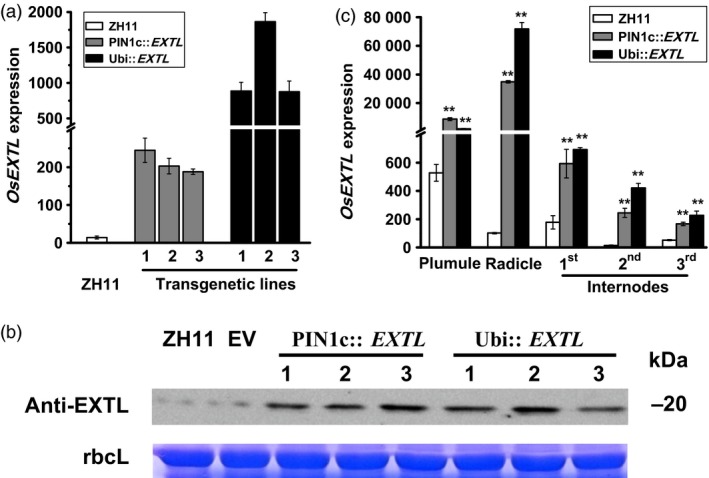
Detection of transgenic rice plants that express *OsEXTL* genes under driven with two distinct promoters (PIN1c, Ubi). (a) Q‐PCR analysis of *OsEXTL* expression levels in three independent homozygous *OsEXTL*‐transgenic lines using the 2nd internodes of stem tissues; ZH11 as cultivar control. (b) Western blotting analysis of OsEXTL protein levels in three independent homozygous *OsEXTL*‐transgenic lines using the 2nd internodes of stem tissues; EV as empty vector; rbcL as rubisco large subunit protein for internal reference from SDS gel running. (c) Q‐PCR analysis of *OsEXTL* gene expression levels in five tissues of the two‐promoter‐driven *OsEXTL*‐transgenic plants; Plumule and radicle collected from 5 days seeding, stems collected at heading stage. All data in (a) and (c) are given as means ± SD (*n* = 3); Student's *t*‐test performed between ZH11 and transgenic plants as **P <* *0.01 and *P <* *0.05.

In this study, we generated transgenic rice plants that expressed *OsEXTL* gene in the background of a rice cultivar (ZH11) using two distinct promoters: PIN1c as a promoter for high gene expression in root, stem‐base and stem tissues of rice (Wang *et al*., 2009) and Ubi as a maize *ubiquitin* promoter for gene overexpression (Figure 2; Table S2). Based on real‐time PCR analysis, three independent homozygous lines of two‐promoter‐driven transgenic plants (PIN1c::*EXTL*, Ubi::*EXTL*) were selected with much higher *OsEXTL* expression levels compared to that in ZH11 (Figure 2a). Western blot analysis further indicated that the three transgenic lines had far higher OsEXTL protein levels than those of the empty vector (EV) and ZH11 (Figure 2b), suggesting that the selected transgenic lines could be used in this study. In addition, we examined variable *OsEXTL* expression in five tissues of transgenic plants, and the Ubi::*EXTL* transgenic plants exhibited relatively higher transcript levels than those of the PIN1c::*EXTL* in four tissues (Figure 2c).

### Reduced plant height and cell length in *OsEXTL*‐transgenic lines

**Figure 3:**
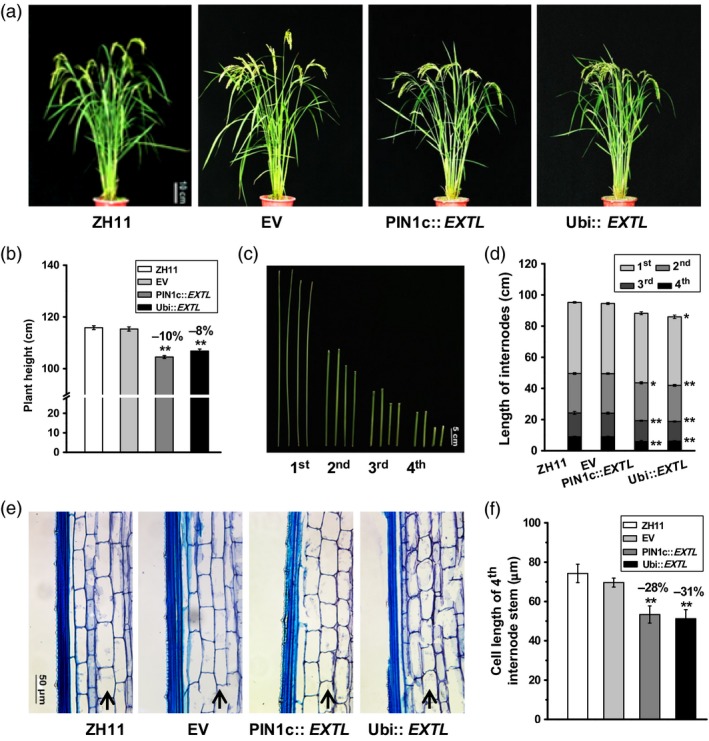
Phenotype observation of *OsEXTL*‐transgenic rice plants. (a) *OsEXTL*‐transgenic plants at filling stage; Scale bar as 10 cm. (b) Plant height at mature stage. (c) Images of four stem internodes in ZH11, EV, PIN1c::*EXTL* and Ubi::*EXTL*; Scale bar as 5 cm. (d) Length of four internodes lengths as observed in (c). (e) Longitudinal section of the 4^th^ internode; Scale bars as 50 μm. (f) Cell length of the 4^th^ internode longitudinal sections as observed in (e). All data in (b), (d) and (f) are given as means ± SD (*n* = 10); Student's *t*‐test between ZH11 and transgenic plants as **P <* *0.01 and *P <* *0.05.

In the field experiment, we observed that both PIN1c::*EXTL* and Ubi::*EXTL* transgenic lines exhibited major agronomic traits, such as grain yield, seed size and total biomass production, similar to those in the ZH11 and EV controls (Table S3). However, the mature *OsEXTL*‐transgenic lines were measured with significantly reduced plant heights from 7% to 10% (*P *<* *0.01), compared to those in the ZH11 and EV (Figure 3a,b; Table S4). In terms of the relatively short plant heights, the PIN1c::*EXTL* transgenic lines exhibited significantly reduced lengths in the flag leaf and the 3^rd^ and 4^th^ stem internodes, whereas the Ubi::*EXTL* transgenic lines had significant decreased lengths in the flag leaf and all four internodes as *P *<* *0.05 and 0.01, respectively (Figure 3c,d; Table S4), indicating a small difference of plant height between the two‐promoter‐driven transgenic plants. To understand the reduced stem length, we measured 28%–31% shorter cell lengths in the 4^th^ internodes of two‐promoter‐driven *OsEXTL‐*transgenic plants compared to those in ZH11 and EV (Figure 3e,f). Hence, the results suggest that overexpression of *OsEXTL* leads to a reduction in cell elongation in stem tissues, yielding relatively short plant heights in transgenic rice.

### Enhanced lodging resistance and mechanical strength in *OsEXTL*‐transgenic lines

In 3‐year (2012, 2013 and 2015) field experiments, we detected the lodging index, a negative factor on lodging resistance, in the *OsEXTL‐*transgenic plants (Table 1). As a result, three independent lines of transgenic plants driven by each promoter exhibited reduced lodging index values from 10% to 48%, compared to those in ZH11 and EV. Notably, despite large variations of lodging index values, the *OsEXTL‐*transgenic lines showed significantly positive correlations among the 3 years experiments as *P *<* *0.01 (Figure S2), indicating that the two‐promoter‐driven *OsEXTL‐*transgenic plants are genetically stable for significantly enhanced lodging resistances. Meanwhile, we measured significantly increased extension and pushing force values in the *OsEXTL‐*transgenic lines compared with those in ZH11 and EV (Table 1), and positive correlations of extension force values were also found among the 3 years experiments with *P *<* *0.01 (Figure S2). Because plant extension and pushing forces are tightly associated with lodging resistance (Berry *et al*., 2004; Hai *et al*., 2005; Hu *et al*., 2017; Zhang *et al*., 2016), this study demonstrated that overexpression of *OsEXTL* could largely enhance plant lodging resistance in transgenic rice plants.

**Table 1:** Detection of lodging index, extension force and pushing force in *OsEXTL*‐transgenic lines in 3‐year field experiments

	Transgenic line	Lodging index						Extension force (*N*)						Pushing force (*N*)	
		2012		2013		2015		2012		2013		2015		2015	
	ZH11	160.83 ± 7.00		170.62 ± 7.66		269.21 ± 8.47		157.58 ± 11.07		180.88 ± 2.21		202.93 ± 7.18		1.66 ± 0.13	
Vector	EV	151.54 ± 10.07		166.70 ± 6.34		278.64 ± 16.1		157.10 ± 8.80		178.96 ± 4.28		210.40 ± 10.84		1.69 ± 0.14	
PIN1c::*EXTL*	1	113.97 ± 5.57**	−29%†	117.03 ± 15.80**	−31%	236.89 ± 15.31**	−12%	189.92 ± 10.99**	+21%	193.21 ± 11.72*	+7%	248.17 ± 12.26**	+22%	2.09 ± 0.09**	+26%
	2	102.36 ± 5.19**	−36%	114.07 ± 11.75**	−33%	240.97 ± 20.04*	−10%	192.85 ± 10.40**	+22%	195.40 ± 12.12*	+8%	217.18 ± 2.51**	+7%	2.10 ± 0.11**	+26%
	3	111.23 ± 8.06**	−31%	116.69 ± 10.27**	−32%	240.45 ± 18.71*	−11%	173.88 ± 9.96*	+10%	188.31 ± 6.86*	+4%	250.00 ± 11.99**	+23%	2.11 ± 0.08**	+27%
Ubi::*EXTL*	1	141.02 ± 13.54**	−12%	147.25 ± 15.08**	−14%	207.64 ± 12.25**	−23%	194.33 ± 4.21**	+23%	213.23 ± 13.51**	+18%	214.67 ± 8.60*	+6%	2.31 ± 0.13**	+39%
	2	111.18 ± 8.98**	−31%	134.77 ± 16.33**	−21%	231.44 ± 10.71**	−14%	197.14 ± 10.86**	+25%	190.06 ± 8.96*	+5%	239.93 ± 10.14**	+18%	2.24 ± 0.11**	+35%
	3	83.39 ± 4.58**	−48%	94.32 ± 8.32**	−45%	151.71 ± 23.06**	−44%	206.33 ± 9.37**	+31%	188.74 ± 6.45*	+4%	253.67 ± 18.12**	+25%	2.49 ± 0.07**	+50%

* and ** indicated significant difference between transgenic lines and ZH11 control by *t*‐test as *P *<* *0.05 and 0.01 (*n* = 10).

^†^Percentage of increased or decreased level between transgenic line and ZH11 by subtraction of two values divided by ZH11.

### Increased secondary cell wall thickness in *OsEXTL*‐transgenic lines

**Figure 4:**
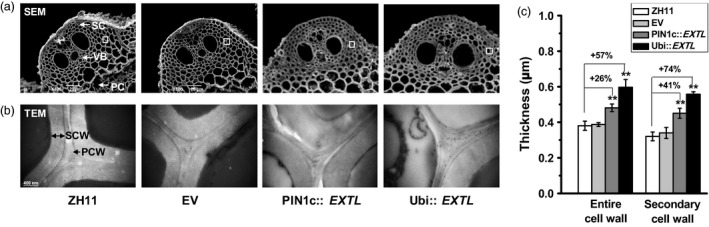
Cell wall observation of *OsEXTL*‐transgenic rice plants. (a) Cell wall image of cross sections of 2nd internode using scanning electron microscopy (SEM); sclerenchyma cells (SC), vascular bundle cells (VB) and parenchyma cells (PC); Scale bars as 20 μm. (b) Cell wall image of sclerenchyma cells using transmission electron microscopy (TEM); PCW as primary cell wall; SCW as secondary cell wall; Scale bars as 200 nm. (c) Quantitative measurement of cell wall thickness as observed by TEM in (b). Data are given as means ± SD (10 cells); Student's *t*‐test between ZH11 and transgenic plants as **P <* *0.01.

As plant cell walls play important roles in plant mechanical strength and morphogenesis, we observed cell wall ultrastructure in the *OsEXTL‐*transgenic plants using scanning electron microscopy (SEM) and transmission electron microscopy (TEM) (Figure 4). Two‐promoter‐driven *OsEXTL‐*transgenic plants exhibited obvious thickened vascular bundle cells (VB), sclerenchyma cells (SC) and parenchyma cells (PC), compared to those in ZH11 and EV (Figure 4a). Notably, the PIN1c::*EXTL* transgenic plants showed increased entire cell wall and secondary cell wall widths by 26% and 41%, respectively, whereas the Ubi::*EXTL* transgenic plants showed increased cell wall and secondary cell wall widths by 57% and 74%, respectively, from the sclerenchyma cells (Figure 4b,c), indicating that overexpression of *OsEXTL* leads to a remarkably increased secondary cell wall thickness in transgenic rice plants.

### Altered cell wall compositions in *OsEXTL*‐transgenic lines

**Figure 5:**
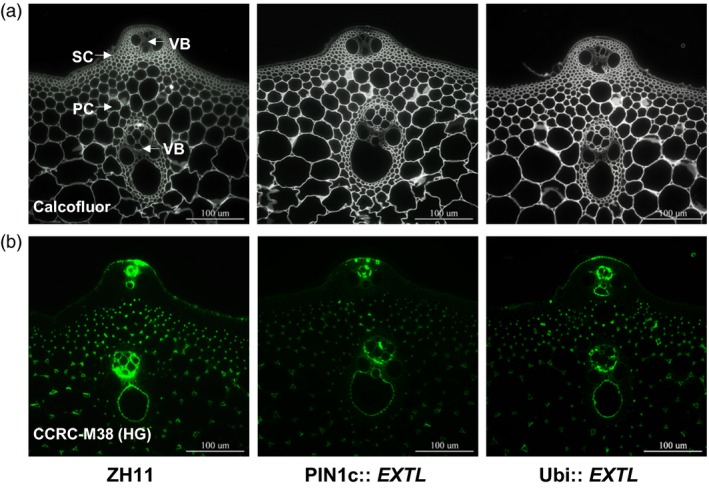
Staining of cellulose and pectin in the 2nd internode tissues of *OsEXTL*‐transgenic rice plants. (a) Calcofluor (white) staining specific for cellulose. (b) Immunohistochemical staining (green) specific for de‐esterified homogalacturonan, using CCRC‐M38 antibody. Scale bars as 100 μm.

To understand the increased secondary cell wall thickness, we determined cell wall compositions in the *OsEXTL‐*transgenic plants. Compared to the ZH11 and EV, three independent lines of PIN1c::*EXTL* transgenic plants were examined with significantly increased cellulose levels by 9%–14% in leaves and 17%–22% in stems. The Ubi::*EXTL* transgenic lines had increased cellulose levels raised by 18%–20% or 14%–25% in leaf or stem tissues, respectively (Table 2), which was confirmed by Calcofluor staining specific for cellulose in the stem tissues (Figure 5a). In contrast, all transgenic lines were determined to have significantly reduced pectin levels by 8%–41%, with *P *<* *0.01. Using a plant glycan‐directed monoclonal antibody, we further observed that the *OsEXTL‐*transgenic plants exhibited much weaker fluorescent signals specific for de‐esterified homogalacturonan of pectin (Figure 5b), confirming the reduced pectin levels in the *OsEXTL‐*transgenic plants. Meanwhile, we determined that the *OsEXTL‐*transgenic plants had hemicelluloses and lignin levels close to those in ZH11 and EV (Table S5). In addition, a similar monosaccharide composition of hemicelluloses was found in both transgenic plants and ZH11 (Table S6). Taken together, it suggests that overexpression of the *OsEXTL* gene could either enhance cellulose deposition into the secondary cell walls or reduce pectin synthesis in primary cell walls in the transgenic plants.

**Table 2:** Cellulose and pectin contents (% dry matter) in leaf and stem tissues of *OsEXTL*‐transgenic lines

	Transgenic line	Leaf				Stem			
		Cellulose		Pectin		Cellulose		Pectin	
	ZH11	18.16 ± 0.09		2.12 ± 0.02		25.18 ± 1.07		1.56 ± 0.03	
Vector	EV	17.72 ± 0.70		2.23 ± 0.07		25.04 ± 0.90		1.57 ± 0.01	
PIN1c::*EXTL*	1	20.79 ± 0.60*	+14%†	1.52 ± 0.05**	−28%	29.40 ± 0.74**	+17%	1.15 ± 0.04**	−26%
	2	19.74 ± 0.47*	+9%	1.94 ± 0.03**	−8%	29.46 ± 0.50**	+17%	1.18 ± 0.04**	−24%
	3	20.14 ± 0.39**	+11%	1.51 ± 0.01**	−29%	30.79 ± 1.53**	+22%	1.22 ± 0.04**	−22%
Ubi::*EXTL*	1	21.57 ± 0.33**	+19%	1.85 ± 0.01**	−13%	31.53 ± 0.38**	+25%	1.21 ± 0.04**	−22%
	2	21.43 ± 0.35**	+18%	1.68 ± 0.04**	−21%	32.25 ± 1.03**	+28%	1.16 ± 0.04**	−26%
	3	21.79 ± 0.72**	+20%	1.70 ± 0.05**	−20%	28.72 ± 0.60**	+14%	0.92 ± 0.02**	−41%

* and ** indicated significant difference between transgenic lines and ZH11 control by *t*‐test as *P *<* *0.05 and 0.01 (*n* = 10).

^†^Percentage of increased or decreased level between transgenic line and ZH11 by subtraction of two values divided by ZH11.

## Discussion

**Figure 6:**
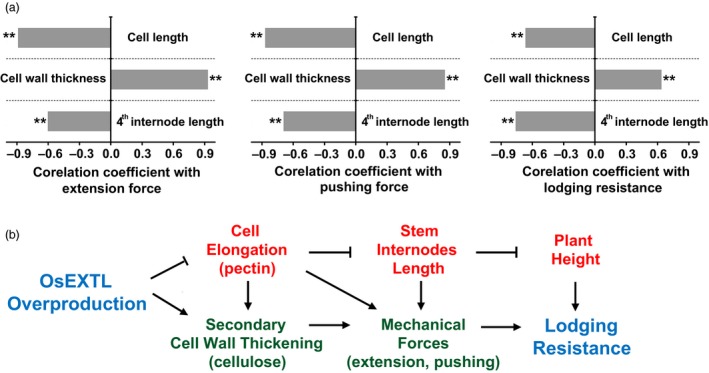
A hypothetical model highlighting OsEXTL large enhancement to plant lodging resistance by reducing cell elongation for short plant heights and increasing cell wall thickness for strong mechanical strength in the transgenic rice plants that overexpress *OsEXTL* gene. (a) Correlation analyses among cell length, cell wall thickness, 4th internode length, mechanical strength and lodging resistance. **indicated significant correlation as *P *<* *0.01 (*n* = 40). (b) Mechanisms that link cell elongation, cell wall thickness and lodging resistance.

Lodging is an important integrated agronomic trait that greatly affects grain yield and quality in rice. Although multiple factors are reportedly associated with lodging features, this study has demonstrated largely improved lodging resistances in the *OsEXTL*‐transgenic rice plants from 3 years field experiments, based on a remarkably reduced lodging index, relatively shortened plant height, and much increased mechanical strength in the transgenic plants as illuminated in the proposed model (Figure 6).

Since plant height is a direct factor negatively accounting for plant lodging resistance (Berry *et al*., 2004), it is understandable that the significantly reduced plant heights should be a major factor in improving lodging resistance in the *OsEXTL*‐transgenic plants. This study provided solid evidences from cell elongation to stem internodes length in supporting for the reduced plant height in the transgenic plants (Figure 6). Because it has been reported that overexpression of *AtEXT1* in *Arabidopsis* leads to a reduction in stem height (Roberts and Shirsat, 2006), the OsEXTL should play a role similar to extensins in down‐regulating cell elongation for plant height control.

Pectin is a major component of primary cell walls and it plays an important role in cell elongation (Iwai *et al*., 2002; Krupková *et al*., 2007). In this work, a remarkably reduced pectin level in the *OsEXTL*‐transgenic plants should be a determining factor of cell elongation and plant height. Because pectin interacts with extensins to form an extensin‐pectate coacervate within plant cell walls (Cannon *et al*., 2008; Lamport *et al*., 2011; Valentin *et al*., 2010), we assumed that the overproduced OsEXTL protein may restrict pectin deposition in the *OsEXTL*‐transgenic plants. In addition, despite that two promoters driving transgenic plants showing significantly different *OsEXTL* expression levels, they both exhibited a reduction of plant height, in particular on the base stem internodes (4^th^) that provide fundamental mechanical strength, leading to a similar enhancement of lodging resistance. This observation also suggests that OsEXTL should be active for regulation of cell elongation and plant growth in the both promoter‐driven transgenic plants.

Plant mechanical strength is another important factor for plant lodging resistance. As plant cell walls basically determine plant mechanical strength and morphogenesis, the increased cell wall thickness (cellulose level) should mainly contribute to the enhanced extension and pushing forces examined in the *OsEXTL*‐transgenic plants. To confirm this, we detected that the cell wall thickness was positively correlated with the mechanical strength (extension and pushing forces) or lodging resistance (*P *<* *0.01) in the transgenic plants (Figure 6a). Notably, we also found that the cell length and the 4^th^ internode length were negatively correlated with mechanical strength and lodging resistance (*P *<* *0.01) in the transgenic plants, providing evidence in support of the proposed model (Figure 6b). Hence, we assumed that the reduced cell elongation may allow an early deposit in the thickened secondary cell walls in the *OsEXTL*‐transgenic plants, whereas the reduced stem internodes should be an additional factor attributing for plant mechanical strength. Surprisingly, although the *OsEXTL*‐transgenic plants contained substantially increased cellulose and reduced pectin, they did not show any significantly altered hemicelluloses or lignin levels, suggesting that the overproduced OsEXTL protein may play a role in maintaining normal cell wall strength and flexibility.

However, although our recently identified rice mutant (*Osfc16*) shows enhanced lodging resistance compared with wild type (Li *et al*., 2017), it has also exhibited different cell wall compositions and features from the *OsEXTL*‐transgenic plants, including thinner cell wall thickness, reduced cellulose crystallinity (CrI) and DP (degree of polymerization) and increased hemicellulose level. Because plant cell walls are of complicated structures and diverse functions with dynamic networks, it remains hard to simply compare impacts of both *OsEXTL*‐transgenic plants and rice *Osfc16* mutant on plant mechanical strength. But the lodging index values should be comparable due to the same approach used for lodging assay in both transgenic plant and mutant.

In addition, despite relatively short plant heights, the *OsEXTL*‐transgenic plants could maintain normal biomass production similar to the ZH11 cultivar and EV, probably due to the increased cellulose levels and cell walls thickness.

In conclusion, this study has for the first time demonstrated OsEXTL enhancement of plant lodging resistance in transgenic rice, and it also indicates potential novel functions of extensins associated with plant growth and development, plant cell wall deposition and wall structural remodelling.

## Experimental procedures

### Genome‐wide expression analysis of *OsEXTL*

The unrooted phylogenetic trees were constructed with the MEGA6 program and the neighbour joining method with 1000 bootstrap replicates (Tamura *et al*., 2013). Expression profile data of rice 33 tissue samples (Table S1) in Minghui63 (MH63) were obtained from CREP database (http://crep.ncpgr.cn), and from a rice transcriptome project using Affymetrix Rice GeneChip microarray (Wang *et al*., 2010).

### Plasmid construction and transformation

The full length of Os*EXTL* cDNA fragment was amplified and inserted into the plant binary vector pCAMBIA1300 (Cambia, Canberra, Australia) driving by two promoters, the rice PIN1c promoter and the maize ubiquitin promoter, respectively. The recombinant constructs were confirmed by sequencing and then introduced into Zhonghua 11 (ZH11, *Oryza sativa* ssp. *japonica*) by *Agrobacterium*‐mediated transformation, with minor modifications (Hiei *et al*., 1994; Lin and Zhang, 2005). Primers of hygromycin gene were designed for PCR analysis. The transgenic lines were assumed as single copy lines with separation rate at about 3 : 1 in *T*_1_ generation, and the homozygous lines were assumed if there was no separation in *T*_2_ and *T*_3_ generation (*n* > 30). For primer detail, see Table S2.

### Agronomic traits evaluation

The transgenic plants harbouring two constructs were generated in ZH11 background. Rice plants were conducted in the normal growing seasons under natural field conditions in the field at Huazhong Agricultural University, Wuhan, China. In all, 10 plants per line were transplanted in a single row with 16 cm between plants and 26 cm between rows. Field management, including irrigation, fertilizer application and pest control, followed essentially the normal agricultural practice. All the plants were grown to mature stage for measuring the agronomic traits. Three homozygous transgenic derived lines (*T*_2_–*T*_4_ progenies) were used for subsequent analysis.

### Total RNA isolation and real‐time PCR

Total RNAs were extracted using Trizol reagent (Invitrogen, Carlsbad, CA) and reverse‐transcribed into cDNA with the GoScript^™^ Reverse Transcription System (Promega, Madison, WI, USA). Quantitative real‐time PCR (qRT‐PCR) was independently performed in triplicate using the SYBR Green PCR Master Mixture (ZF101, ZOMANBIO, Beijing, China). A rice *polyubiquitin* gene (*OsUBQ1*) was used as the internal control. Primers used in this study are listed in Table S2.

### OsEXTL antibody preparation

Antibody preparation was performed as described previously (Li *et al*., 2013a). Prediction of the areas of OsEXTL high antigen epitope was done by online software (http://imed.med.ucm.es/Tools/antigenic.pl). The regions encoding the first hypervariable region of OsEXTL was amplified by PCR. The amplified sequences were constructed into pGEX4T‐3 vector in frame with a GST tag, and the recombinant peptides were induced in *Escherichia coli* BL21. The purified peptides were injected into rabbits and antibodies were prepared by Nanjing GenScript Corporation (Nanjing, China).

### Western blot

Total proteins were extracted with extraction buffer containing protease inhibitors and separated by 12% SDS‐PAGE. The following procedures were performed as described by Li *et al*. (2017). The rubisco large subunit protein (rbcL) of SDS‐PAGE gel was regarded as internal reference

### Microscope observation

The sample preparation was performed as previously described (Cao *et al*., 2014). The second and forth internodes (1 cm above the node) at the heading stage were cut pieces, subsequently fixed with 4% (w/v) paraformaldehyde, and dehydrated through an ethanol gradient (30%, 50%, 70%, 90%, 100% and 100%, each for 30 min), and then embedded in paraplast plus. The sections (8 μm thickness) were cut using a microtome (RM2265, Leica, Leica Microsystems, Nussloch, Germany) and placed on lysine‐treated slides which were dried for 2 days at 37 °C, and de‐waxed with xylene and hydrated through an ethanol series (100%–0%).

The second internode at the heading stage was used for analysing the distribution of cell wall polysaccharides. The sections were treated with PBS buffer contained 3% SMP (skim milk powder, w/v) for 1 h, and incubated with PBS containing 10 μg/mL CCRC‐38 (de‐esterified homogalacturonan) for another 1 h. The immunolabelled samples were washed three times (5 min each) with PBS and incubated with a 100‐fold dilution of anti‐mouse‐IgG in dark for 2 h. The anti‐mouse‐IgG antibody was labelled by fluorescein‐isothiocyanate (FITC). Counterstaining was performed with calcofluor white M2R fluorochrome (fluorescent brightener 28; Sigma; 0.25 μg/mL in dH_2_O). Immunofluorescence sections were imaged using a microscope (Olympus BX‐61, Olympus, Tokyo, Japan) equipped with the following filter sets: 350/450 nm (ex/em) for visualizing calcofluor white stained cell walls, and 490/520 nm (ex/em) for green emission of the FITC fluorochrome, respectively.

The forth internode at the heading stage was used for longitudinal sections to quantification the cell size. Sections were stained with toluidine blue and then photographed under a microscope (Olympus BX‐61).

### Scanning electron microscopy and transmission electron microscopy analyses

The second internode at the heading stage was cut into 1–2 mm pieces subsequently fixed with 2.5% (v/v) glutaraldehyde, vacuumed three times, and fixed for at least 24 h. Samples were natural dried, sputter‐coated with gold particles, observed and photographed using a scanning electron microscope (JSM‐6390LV; JEOL, Tokyo, Japan). Scanning electron microscopy (SEM) analysis was based on at least three biological replications of the mounted specimens. All procedures were carried out according to the manufacturer's protocol.

Transmission electron microscopy (TEM) was used to observe cell wall structures in the third leaf veins of three‐leaves‐old seedlings. Tissues were high‐pressure frozen, freeze substituted, embedded, sectioned and viewed according to McFarlane *et al*. (2008). The samples were post‐fixed in 2% (w/v) OsO_4_ for 1 h after extensively washing in the PBS buffer and embedded with Suprr Kit (Sigma‐Aldrich, St. Louis, MO, USA). Sample sections were cut with an Ultracut E ultrami‐crotome (Leica) and picked up on formvar‐coated copper grids. After post‐staining with uranyl acetate and lead citrate, the specimen was viewed under a Hitachi H7650 (Hitachi Ltd., Tokyo, Japan) transmission electron microscope. The width of cell wall was measured using the software ImageJ (NIH), and more than 20 cell walls each for the different genotypes were measured. Significance was estimated using Student's *t* test.

### Plant mechanical properties measurement

Plant lodging index was detected as previously described (Li *et al*., 2015, 2017) using the stem tissues at 30 days after flowering with 10 independent biological repeats. The breaking resistance of the third internode was detected using a Prostrate Tester (DIK 7401, Daiki Rika Kogyo Co., Ltd., Tokyo, Japan), with the distance between fulcra of the tester at 5 cm. Fresh weight (W) of the upper portion of the plant was measured including panicle and the three internodes, leaf and leaf sheath. Bending moment (BM) and lodging index (LI) were calculated using the following formula: BM = Length from the third internode to the top of panicle × W, LI = BM/breaking resistance.

The extension force was tested at the milk maturity stage. The stems were cut into segments of 5 cm in length. The stretching force of the samples before being broken was measured with a universal force/length testing device (model RH‐K300, Guangzhou, China) (Zhang *et al*., 2016). The newton is used as the unit of extension force (EF).

The pushing force (PF), a parameter for stem strength, was measured when the plant was pushed to an angle of 45° from the vertical at the milk stage according to the method described by Hai *et al*. (2005). Five stems were measured in each experimental unit (plot) by the prostrate tester (DIK‐7400, Daiki Rika Kogyo Co. Ltd., Tokyo Japan). The instrument functions were on the basis of the principle of action and reaction. The newton is used as the unit of PF.

### Cellulose, pectin and hemicelluloses determination

The plant tissues were dried at 65 °C until a constant weight was reached, and mechanical crushed using a knife‐mill. Plant cell wall fractionations were extracted as described previously (Li *et al*., 2015; Peng *et al*., 2000) with minor modifications.

For crystalline cellulose extraction, samples (0.1 g) were suspended in 5.0 mL acetic acid–nitric acid–water (8 : 1 : 2, v/v/v) and heated for 1 h in a boiling water bath with stirring every 10 min. After centrifugation, the pellet was washed several times with 5.0 mL water and dissolved in 67% H_2_SO_4_. Total hexoses in 67% H_2_SO_4_ were regarded as cellulose.

For pectin extraction, the dry biomass powder samples (0.1 g) were treated by potassium phosphate buffer (pH 7.0), chloroform–methanol (1:1, v/v) and DMSO–water (9:1, v/v) to remove soluble sugar, lipids and starch. The remaining pellets as crude cell wall was suspended in 0.5% (w/v) ammonium oxalate (5.0 mL) and heated for 1 h in a boiling water bath, and the supernatants were total pectin.

For hemicelluloses monosaccharide analysis, the pellet after pectin extraction was dissolved by 1.0 mL 2 m TFA to release free monosaccharides in the sealed tube at 121 °C in autoclave (15 psi) for 1 h. The supernatants extracted from TFA reaction were separately transferred into 5 mL screw‐cap test tubes. Myo‐inositol (200 μg) was added as the internal standard. The supernatant was dried under vacuum at 38 °C to remove TFA, then were neutralized, dialysed and lyophilized according to the method described by Xu *et al*. (2012).

### Colorimetric assay of hexoses and pentoses

The hexose and pentose assays were performed using an UV/VIS spectrometer for the absorbance reading according to Li *et al*. (2013b) (V‐1100D, Shanghai MAPADA Instruments Co., Ltd., Shanghai, China). Total hexoses were measured by the anthrone/H_2_SO_4_ method and absorbance reading at 620 nm (Fry, 1988). Total pentoses were detected using the orcinol/HCl method and absorbance reading at 660 nm (Dische, 1962). The standard curves for hexoses and pentoses were drawn using D‐glucose and D‐xylose as standard. As the high pentose level affects the absorbance reading at 620 nm for the hexose assay by the anthrone/H_2_SO_4_ method, the deduction from pentoses was carried out for a final hexose calculation. All experiments were carried out in biological triplicate.

### Hemicellulose monosaccharide analysis by GC‐MS

GC/MS analysis was conducted with SHIMADZU GCMSQP2010 Plus according to Xu *et al*. (2012). L‐rhamnose, L‐arabinose, L‐fucose, D‐xylose, D‐galactose, D‐glucose and D‐mannose were used as monosaccharide standards obtained from Sinopham Chemical Reagent Co., Ltd. The GC‐MS analytical conditions: Restek Rxi‐5 ms, 30 m × 0.25 mm ID × 0.25 μm df column. Carrier gas: He. Injection Method: Split. Injection port: 250 °C, Interface: 250 °C. Injection Volume: 1.0 μL. The temperature program: from 170 °C (held for 12 min) to 220 °C (held for 8 min) at 3 °C/min. Ion source temperature: 200 °C, ACQ Mode: SIM. The mass spectrometer was operated in the EI mode with ionization energy of 70 ev. Mass spectra were acquired with full scans based on the temperature program from 50 to 500 m/z in 0.45 s. Calibration curves of all analytes routinely yielded correlation coefficients 0.999 or better. Peaks were identified by mass profiles and/or retention times of standards. Monosaccharides were quantified based on standard curves.

### Total lignin measurement

Total lignin was determined by two‐step acid hydrolysis method according to Laboratory Analytical Procedure of the National Renewable Energy Laboratory (Sluiter *et al*., 2008), as described by Wu *et al*. (2013). All samples were carried out in triplicate.

### Statistical analyses

Both two‐tailed Student's *t*‐test and analysis of variance (ANOVA) were performed with SPSS. Significance was accepted at the levels of *P *<* *0.05 and *P *<* *0.01. Correlation coefficients were calculated by performing Spearman rank correlation analysis for all pairs of measured traits across the whole population.

## Supporting information


**Figure S1** Phylogenetic trees of OsExtensins family. Red frame as OsExtensin‐like protein.
**Figure S2** Correlation analysis of lodging index and extension force among three years field experiments.
**Table S1** Tissues and developmental stages throughout the rice life cycles.**Table S2** Primers for vector construction and real‐time PCR.
**Table S3** Major agronomic traits of *OsEXTL*‐transgenic lines in field experiment.
**Table S4** Plant height and lengths of flag leaf and internodes at mature transgenic plants in field experiment.
**Table S5** Hemicelluloses and lignin content (% dry matter) in leaf and stem.
**Table S6** Monosaccharide composition of hemicelluloses (%).
