# Supplementary material for: Ectopic expression of a novel OsExtensin‐like gene consistently enhances plant lodging resistance by regulating cell elongation and cell wall thickening in rice
Source: Plant Biotechnol J. 2017 Jul 15;16(1):254–63. doi: 10.1111/pbi.12766 (PMC5785348; doi:10.1111/pbi.12766)
Supplement: Supplementary file 1 — Figure S1 Phylogenetic trees of OsExtensins family. Red frame as OsExtensin‐like protein. Figure S2 Correlation analysis of lodging index and extension force among three years field experiments. Table S1 Tissues and developmental stages throughout the rice life cycles.Table S2 Primers for vector construction and real‐time PCR. Table S3 Major agronomic traits of OsEXTL‐transgenic lines in field experiment. Table S4 Plant height and lengths of flag leaf and internodes at mature transgenic plants in field experiment. Table S5 Hemicelluloses and lignin content (% dry matter) in leaf and stem. Table S6 Monosaccharide composition of hemicelluloses (%). [file PBI-16-254-s001.pdf]

Supplementary Figure 1. Phylogenetic trees of OsExtensins family. Red frame as OsExtensin-like protein.

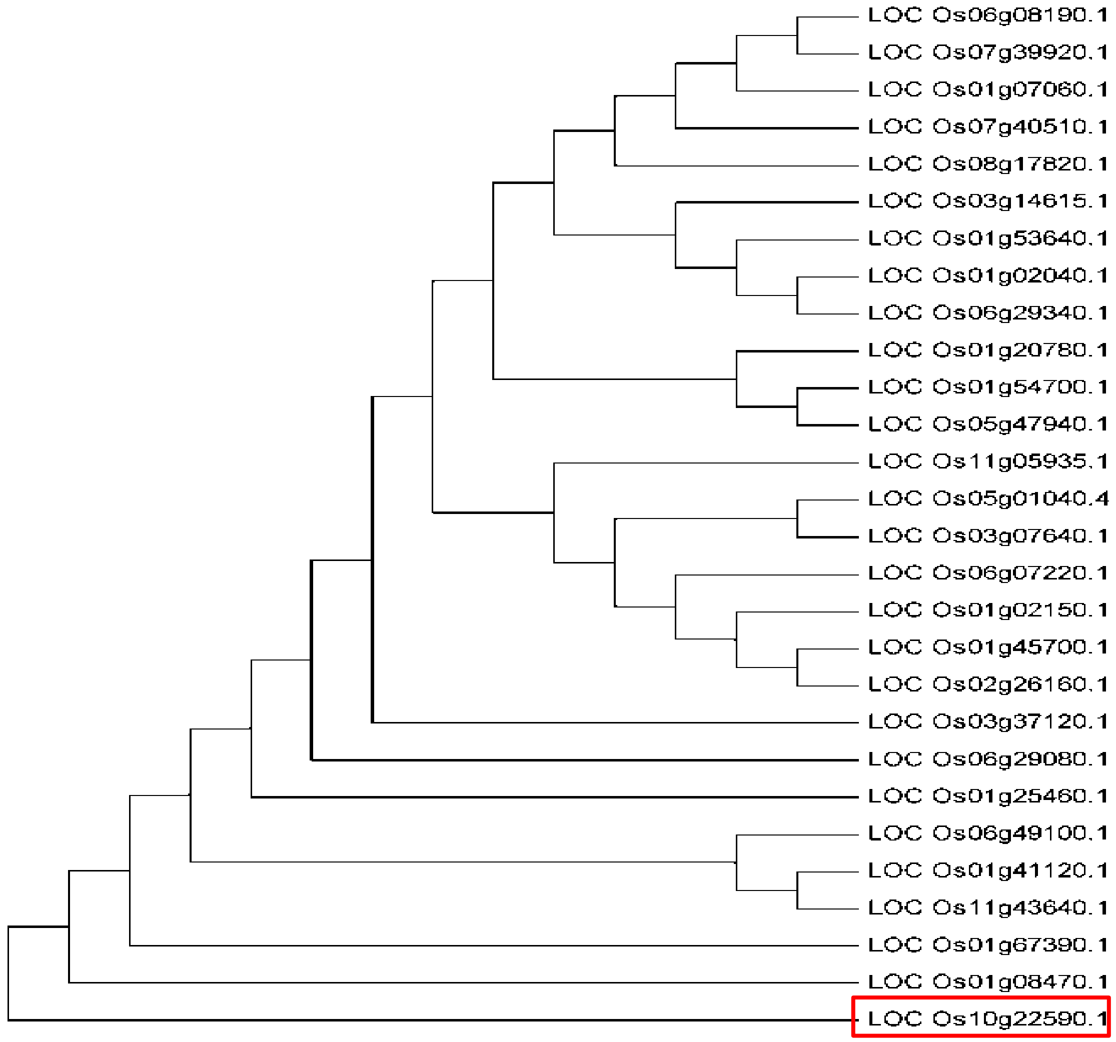

Supplementary Figure 2. Correlation analysis of lodging index and extension force among three years field experiments.

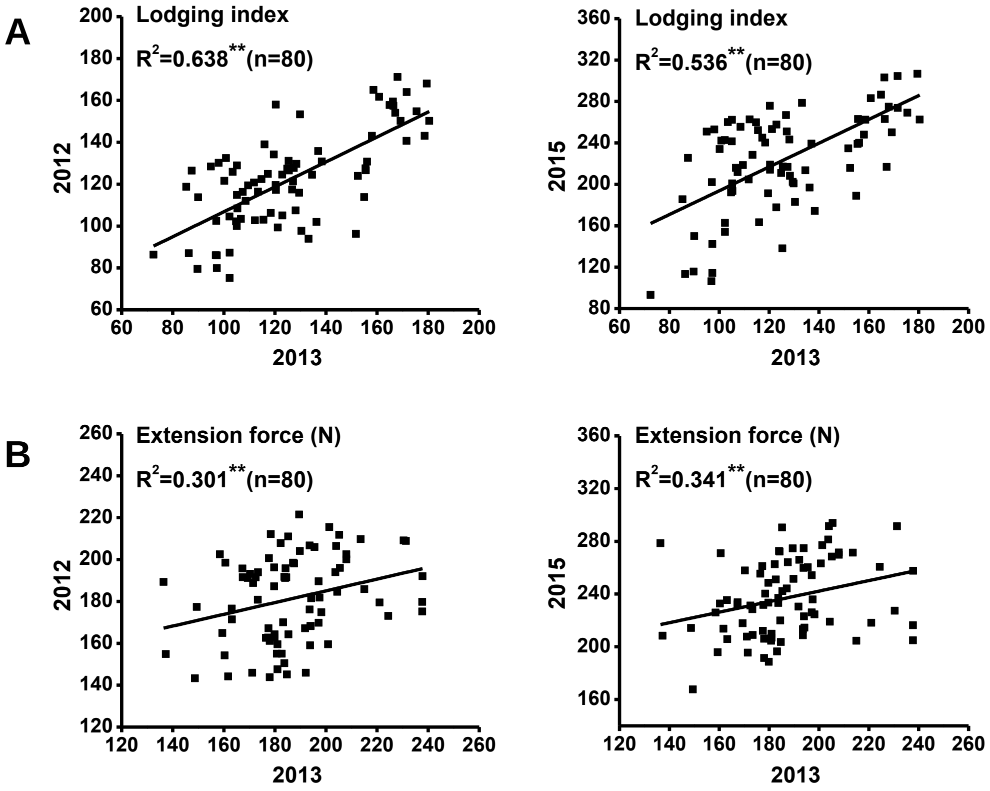

**, Indicated significant correlation as $P < 0.01$ (n = 80)

Supplemental Table 1. Tissues and developmental stages throughout the rice life cycles

Sample No	Abbreviation	Tissues or organ	Developmental stages
1	Calli 1	Calli	15 DAS (days after subculture)
2	Calli 2	Calli	5 DAR (days after regeneration)
3	Calli 3	Calli	Screening stage
4	Calli 4	Calli	15 DAI T2 (days after induction)
5	Calli 5	Calli	15 DAI T3 (days after induction)
6	Seed imbibition	Seed	72 h after imbibition
7	Seed germination	Embryo bud and radicle	3 days after germination
8	Plumule/Dark	Plumule	48 hours after emergence, Dark
9	Plumule/Light	Plumule	48 hours after emergence, Light
10	Radicle/Dark	Radicle	48 hours after emergence, Dark
11	Radicle/Light	Radicle	48 hours after emergence, Light
12	Seedlings	Seedling	Three-leaf stage
13	Young shoot	Shoot	Seedlings with 2 tillers
14	Young root	Root	Seedlings with 2 tillers
15	Mature leaf	Leaf	Stage 3(secondary branch primordium differentiation stage)
16	Old leaf	Leaf	4– young panicle
17	Mature sheath	Sheath	Stage 3
18	Old sheath	Sheath	4– young panicle
19	Young flag leaf	Flag leaf	5 DBH (5 days before heading)
20	Old flag leaf	Flag leaf	14 DAH (14 days after heading)
21	Panicle, stage 3	Young Panicle	Stage 3
22	Panicle, stage 4	Young Panicle	Stage 4 (pistil/stamen primordium differentiation stage)
23	Panicle, stage 5	Young Panicle	Stage 5 (pollen-mother cell formation stage)
24	Young panicle	Panicle	4– young panicle
25	Old panicle	Panicle	Heading stage
26	Young stem	Stem	5 DBH
27	Old stem	Stem	Heading stage
28	Hull	Hull	1 DBF (1 day before flowering)
29	Spikelet	Spikelet	3 DAP (3 days after pollination)
30	Stamen	Stamen	1 DBF
31	Endosperm 1	Endosperm	7 DAP (7 days after pollination)
32	Endosperm 2	Endosperm	14 DAP (14 days after pollination)
33	Endosperm 3	Endosperm	21 DAP (21 days after pollination)

Supplemental Table 2. Primers for vector construction and real-time PCR.

Genes	Purpose	Primer name	Primer sequence (5'-3')	Enzyme	TM(℃)
OsPIN1c	Promoter clone	Os*PIN1c*-F	GCTCTAGATGATCACAGGATCTGTACTCT	Xba I	56
		Os*PIN1c*-R	CGGGATCCGGCTGCCGATGCAAGAGGCTT	BamH I	
OsEXTL	*OsEXTL* under PIN1c promoter	OsEXTL-F	CGCGGATCCACGACCATTATTCCTCCTCCTC	BamH I	54
		OsEXTL-R	GCCTGATAGAAAGCGACAACATG		
	OsEXTL under Ubi promoter	OsEXTL-F	CGCGGATCCACGACCATTATTCCTCCTCCTC	BamH I	54
		OsEXTL-R2	CGGGGTACCGCCTGATAGAAAGCGACAACATG	Kpn l	
	OsEXTL antibody	GEXTL-F	CGGGATCCGGAGCGAAGGTAAAGGTTGA	BamH I	58
		GEXTL-R	GGAATTCCATCGTTGACAGGGATGTTGTA	EcoR I	
	Q-PCR	QEXTL-F	GGCTCCTGTCTTCCTCACCA		60
		QEXTL-R	CGTCCACTTCCTCGTACATCTT		
OsUBQ1	Q-PCR	OsUBQ1-F	CAGTAAGTCCTCAGCCAT		60
		OsUBQ1-R	AGACCAGACAACCATAGC		

Supplemental Table 3. Major agronomic traits of *OsEXTL*-transgenic lines in field experiment.

	Transgenic line	Grain yield (g/plant)	Biomass production (g/plant)	1,000-grain weight (g)	Tillers per plant	Grains per panicle	Seed setting rate(%)
Vector	ZH11	28.65±1.55	28.58±4.93	22.92±0.68	13.50±1.05	129.33±4.18	80.58±2.94
	EV	28.44±2.21	29.95±3.37	22.83±1.06	14.00±0.89	131.33±8.64	81.50±2.04
PIN1c ::*EXTL*	1	28.10±2.14	29.62±2.93	23.42±0.54	13.00±1.41	141.75±14.82	87.79±3.31**
	2	30.21±1.97	29.53±4.04	23.32±0.46	14.67±0.58	136.00±15.14	82.52±2.43
	3	28.37±1.31	30.33±2.93	23.52±1.16	13.33±1.15	136.75±16.07	83.45±4.46
Ubi:: *EXTL*	1	28.42±1.68	30.79±3.18	23.33±0.41	13.50±0.84	140.50±14.53	87.05±3.19**
	2	29.99±2.04	30.69±3.91	23.30±0.62	13.80±1.64	142.33±12.13	85.83±1.93*
	3	27.75±3.16	31.42±5.63	23.32±0.53	13.80±1.48	134.50±10.52	86.03±2.15*

* and **, Indicated significant difference between transgenic lines and ZH11 control by *t*-test as $P < 0.05$ and 0.01.
All data are given as means ± SD (n = 10)

Supplemental Table 4. Plant height and lengths of flag leaf and internodes at mature transgenic plants in field experiment.

	Transgenic line	Plant height (cm)		Flag leaf (cm)		Internodes (cm)							
						1st		2nd		3rd		4th	
	ZH11	115.83±0.74		34.25±0.52		45.83±0.41		25.33 ±0.61		15.33±0.98		8.92±0.38	
Vector	EV	115.33±0.82		33.67±1.03		45.00±0.63		25.42±0.49		15.08±0.66		9.00±0.32	
PIN1c:: *EXTL*	1	105.17±0.75**	-9%[@]	31.33±1.37**	-9%	45.57±1.79		25.17±0.98		12.75±0.27**	-17%	5.92±0.38**	-34%
	2	104.50±0.55**	-10%	32.75±0.76**	-4%	44.67±0.98		24.33±0.68*	-4%	13.42±0.38**	-12%	5.83±0.82**	-35%
	3	105.67±0.82**	-9%	34.83±0.98		45.00±0.63		24.17±0.75*	-5%	14.25±0.27*	-7%	5.33±0.26**	-40%
Ubi:: *EXTL*	1	107.17±0.75**	-7%	32.00±0.32**	-7%	44.83±0.41*	-2%	22.75±0.61**	-10%	12.58±0.38**	-18%	6.17±0.26**	-31%
	2	107.00±0.63**	-8%	27.33±0.52**	-20%	44.00±1.10*	-4%	23.17±0.61**	-9%	12.67±0.41**	-17%	6.08±0.38**	-32%
	3	106.83±0.75**	-8%	28.33±0.98**	-17%	44.33±0.88*	-3%	23.00±0.45**	-9%	12.58±0.86**	-18%	6.67±0.52**	-25%

* and **, Indicated significant difference between transgenic lines and ZH11 control by *t*-test as $P < 0.05$ and 0.01 (n = 10);

[@] Percentage of increased or decreased level between transgenic line and ZH11 by subtraction of two values divided by ZH11.

Supplemental Table 5. Hemicelluloses and lignin content (% dry matter) in leaf and stem

	Transgenic line	Leaf	Stem	
		Hemicelluloses	Hemicelluloses	Lignin
Vector	ZH11	17.98±0.37	21.00±0.23	13.00±0.15
	EV	17.58±0.21	21.24±0.43	12.83±0.42
PIN1c::*EXTL*	1	17.66±0.14	20.58±0.29	13.13±0.64
	2	17.54±0.04	22.24±0.53*	12.94±0.57
	3	18.37±0.53	20.43±0.31	13.45±0.75
Ubi::*EXTL*	1	18.16±0.44	22.06±0.55	13.16±0.85
	2	17.58±0.24	20.28±0.39	12.84±0.61
	3	18.86±0.37*	22.04±0.57	12.97±0.76

*****Indicated significant difference between transgenic lines and ZH11 control by *t*-test as $P < 0.05$. All data are given as means ± SD (n = 3)

Supplemental Table 6. Monosaccharide composition of hemicelluloses (%)

	Transgenic line	Leaf						Stem					
		Rha	Fuc	Ara	Xyl	Man	Gal	Rha	Fuc	Ara	Xyl	Man	Gal
Vector	ZH11	0.34	0.03	13.79	82.57	0.16	3.11	0.22	0.02	6.39	92.07	0.06	1.24
	EV	0.40	0.04	13.22	83.06	0.16	3.12	0.20	0.01	6.12	92.40	0.05	1.23
PIN1c::*EXTL*	1	0.30	0.04	13.23	82.66	0.09	3.69	0.19	0.01	7.10	91.18	0.06	1.47
	2	0.49	0.09	15.39	80.72	0.10	3.22	0.15	0.01	6.81	91.46	0.06	1.51
	3	0.29	0.02	13.99	82.25	0.10	3.33	0.23	0.01	6.53	91.79	0.06	1.38
Ubi::*EXTL*	1	0.37	0.04	14.64	81.17	0.10	3.67	0.37	0.04	6.64	88.97	0.11	3.87
	2	0.39	0.04	13.54	81.98	0.08	3.98	0.19	0.01	6.31	92.02	0.05	1.41
	3	0.46	0.06	14.62	80.69	0.08	4.10	0.20	0.01	6.37	91.96	0.05	1.40
